# Supplementary material for: Hepatoprotective Effect of Kombucha Tea in Rodent Model of Nonalcoholic Fatty Liver Disease/Nonalcoholic Steatohepatitis
Source: Int J Mol Sci. 2019 May 13;20(9):2369. doi: 10.3390/ijms20092369 (PMC6539514; doi:10.3390/ijms20092369)
Supplement: Supplementary file 1 [file ijms-20-02369-s001.pdf]

Supplementary Materials to

Hepatoprotective Effect of Kombucha Tea in Rodent Model of Nonalcoholic Steatohepatitis

Chanbin Lee, Jieun Kim, Sihyung Wang, Sumi Sung, Namgyu Kim, Hyun-Hee Lee, Young-Su Seo, and Youngmi Jung

Table of Contents

Supplementary Table …………………………………………………………………..…… 2

Supplementary Figure ………..…………..……………………………..…………………….….. 4

Supplementary Table

Table S1. Changes in liver weight and body weight in experimental animal.

	**db/db Mice**		
Parameter	CON	M + V	M + K
Liver weight (g)	1.916 ± 0.177	1.304 ± 0.044*	1.404 ± 0.071*
Body weight (g)	29.810 ± 3.530	24.992 ± 0.615**	27.933 ± 0.933**/#
LW/BW ratio	0.065 ± 0.002	0.052 ± 0.001**	0.500 ± 0.002**

Liver weight (LW), Body weight (BW), and the ratio of liver to body weight (LW/BW) of CON, M+V and M+K groups. (*$p<0.05$, **$p<0.005$ vs CON group, #$p<0.05$, ##$p<0.005$ vs M+V group)

Table S2. Mouse primer sequences used for QRT-PCR

GENE	Forward	Reverse
G6pc	5'-TCCTCCTCAGCCTATGTCTGCATTC-3'	5'-GAGAGAAGAATCCTGGGTCTCCTTG-3'
9s	5'-GGGCCTGAAGATTGAGGATT-3'	5'-CGGGCATGGTGAATAGATTT-3'
Cd36	5'-TCCTCTGACATTTGCAGGTCTATC-3'	5'-AAAGGCATTGGCTGGAAGAA-3'
Pparγ	5'-CACAATGCCATCAGGTTTGG-3'	5'-GCTGGTCGATATCACTGGAGATC-3'
Fas	5'-GCTGCGGAAACTTCAGGAAAT-3'	5'-AGAGACGTGTCACTCCTGGACTT-3'
Srebp1c	5'-GGAGCCATGGATTGCACATT-3'	5'-GGCCCGGGAAGTCACTGT-3'
Dgat2	5'-CTGGCTGATAGCTGCTCTCTACTTC-3'	5'-TGTGATCTCCTGCCACCTTTC -3'
Fabp1	5'-CCAGGAGAACTTTGAGCCATTC-3'	5'-TGTCCTTCCCTTTCTGGATGA -3'
Pppargc1α	5'-CAGAGGATGGACACTGTAAAGG-3'	5'-CGGCACTTCTTGATCAAGCC-3'
Pparα	5'-CAGGAGAGCAGGGATTTGCA-3'	5'-CCTACGCTCAGCCCTCTTCAT-3'
Aox1	5'-CAGGAAGAGCAAGGAAGTCC-3'	5'-CCTTTCTGGCTGATCCCATA-3'
Cpt1	5'-CAGAGGATGGACACTGTAAAGG-3'	5'-CGGCAGTTCTTGATCAAGCC-3'
Mcad	5'-GATGCATCACCCTCGTGTAAC-3'	5'-AAGCCCTTTTCCCCTGAAG-3'
Cxcl1	5'-CCCAAACCGAAGTCATAGCC-3'	5'-TCAGAAGCCAGCGTTCACC-3'
Cxcl2	5'-GCCCAGACAGAAGTCATAGCC-3'	5'-TTCTCTTTGGTTCTTCCGTTGA-3'
Il-1β	5'-ACTCCTTAGTCCGCCA-3'	5'-TGGTTTCTTGTGACCCTGAGC-3'
Tnfα	5'- TCGTAGCAAACCACCAAGTG-3'	5'- ATATAGCAAATCGGCTGACG -3'
Shh	5'-CTGGCCAGATGTTTTCTGGT-3'	5'-GATGTCGGGGTTGTAATTGG-3'
Smo	5'-CAGCAAGATCTTCGAGACCA-3'	5'-AAGTGGCAGATGAAGGTGAT-3'
Gli2	5'-CAAGCAGAACAGCGAGTCAC-3'	5'-CCTCAGCCTCAGTCTTGACC-3'
Tgfβ	5'-TTGCCCTCTACAACCAACACAA-3'	5'-GGCTTGCGACCCACGTAG-3'
α-sma	5'-AAACAGGAATACGACGAAG-3'	5'-CAGGAATGATTTGGAAAGGA-3'
Col1α1	5'-GAGCGGAGAGTACTGGATCG-3'	5'-GCTTCTTTTCCTTGGGGTTC-3'
Ctgf	5'-ACTGCCCCTTCCCGAGAA-3'	5'-TCCTTGGGCTCGTCACACA-3'
Timp1	5'-CCTTGCAAACTGGAGAGTGACA-3'	5'-AAGCAAAGTGACGGCTCTGGT-3'

Supplementary Figure

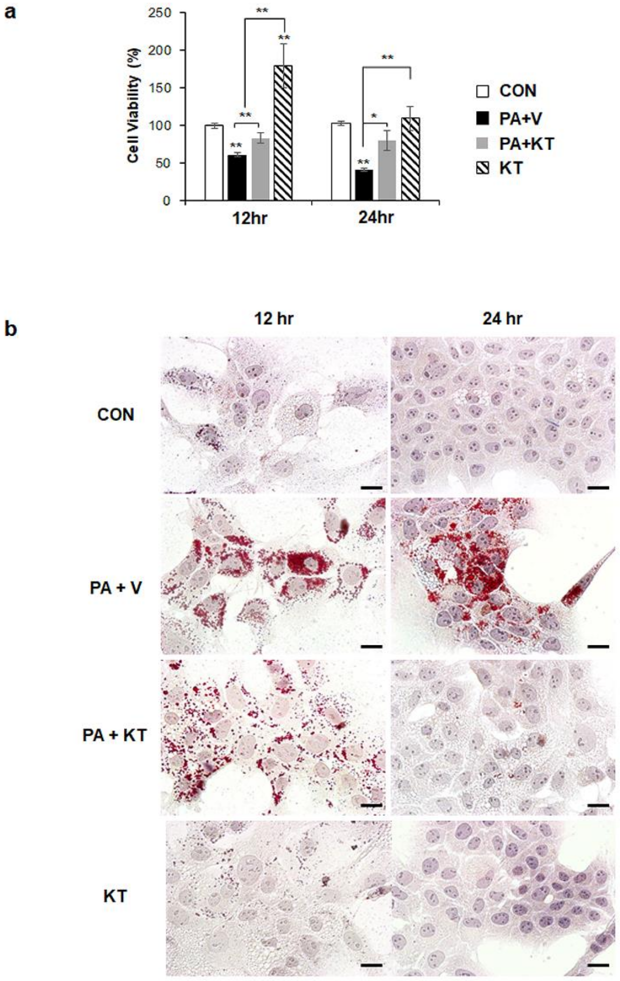

Figure S1 KT increases viability of and suppress lipid accumulation in PA-injured AML12

(a) Cell viability of AML12, normal hepatocyte cell line originated from mouse, treated with KT was analyzed using MTS assay. After being exposed to 250μM of palmitate (PA) for 24hours, primary hepatocytes were treated with vehicle (PA + V) or KT (PA+KT) for 12 and 24 hours. As a control, AML12 was treated with equal volume of vehicle (BSA) without PA for 24 hours, and then given with (KT) or without KT (CON). The mean±SEM results obtained from three repetitive experiments are graphed (**p*<0.05, ***p*<0.005 vs CON). (b) Oil red O staining in these cells. Representative images are shown (Scale bars: 20μm)
